# Expression of Autophagy-Related Proteins in Hürthle Cell Neoplasm Is Different from That in Follicular Neoplasm

**DOI:** 10.1155/2017/1372387

**Published:** 2017-07-27

**Authors:** Yoon Jin Cha, Hye Min Kim, Ja Seung Koo

**Affiliations:** Department of Pathology, Yonsei University College of Medicine, Seoul, Republic of Korea

## Abstract

**Purpose:**

We aimed to evaluate expression of autophagy-related proteins in Hürthle cell neoplasm (HCN) and follicular neoplasm (FN) and assess the clinical implications.

**Methods:**

265 FNs (112 follicular carcinomas and 153 follicular adenomas) and 108 HCNs (27 Hürthle cell carcinomas and 81 Hürthle cell adenomas) were made into a tissue microarray. Immunohistochemical staining and Western blot for autophagy-related proteins (beclin-1, light chain (LC) 3A, LC3B, p62, and BNIP3) were performed, and the results were statistically analyzed.

**Results:**

A higher expression rate of beclin-1, LC3B, p62, and BNIP3 was found in HCN than in FN (*P* < 0.001). The expression rate of beclin-1, LC3B, p62, and BNIP3 was the highest in HCCs followed by HCAs, FCs, and FAs in that order (*P* < 0.001). HCCs were positive for the largest number of autophagy-related proteins followed by HCAs, FCs, and FAs (*P* < 0.001), and most of the positive markers identified in HCCs were the high autophagy type (*P* < 0.001), defined by positive staining for three or more of the five autophagy-related proteins.

**Conclusion:**

The autophagy-related proteins, beclin-1, LC3A, LC3B, p62, and BNIP3, were more frequently expressed in HCNs than in FNs, and HCCs showed the highest expression rate.

## 1. Introduction

Autophagy is the lysosomal disassembly of cellular components and is separated into microautophagy, chaperone-mediated autophagy, and macroautophagy subtypes. Macroautophagy is the major type of autophagy, and its underlying process has been investigated extensively. Autophagy is the process of removing and recycling dysfunctional or damaged cellular components, and it plays an important homeostatic role [1–4]. Autophagy activity is measured by the expression of several surrogate proteins involving the autophagy process: beclin-1 in nucleation [5–8], LC (light chain) 3 in elongation and generation of the autophagosome [9–11], scaffold protein p62 in transportation of ubiquitinated proteins to the autophagosome [12, 13], and BNIP3 involving mitophagy [14].

Autophagy plays a significant role in both tumor and normal cells. Cancer cells survive via angiogenesis and/or aerobic glycolysis in harsh environments including hypoxia and decreased nutrients. Highly aggressive malignant tumor cells use alternative metabolic pathways to provide energy via autophagy and to recycle cytoplasmic components in order to meet high metabolic demands [15, 16]. However, unrestrained autophagy results in progressive consumption of cellular constituents and subsequent cellular death [17, 18].

Hürthle cell neoplasm (HCN) of the thyroid is a variant of follicular neoplasm (FN). Hürthle cell adenoma (HCA) comprises 10%–15% of follicular adenomas (FA), and Hürthle cell carcinoma (HCC) accounts for 20%–25% of follicular carcinomas (FC) [19, 20]. Hürthle cells originate from follicular epithelial cells and are characterized by ample, granular cytoplasm and prominent nucleoli [21]. In the current WHO classification, HCN is categorized as a variant of FN [22]. However, there is debate as to whether HCN is a disease entity distinct from FN owing to its association with nodal metastasis, higher recurrence rate and disease-related mortality [23, 24], and frequent TERT C228T promoter mutation [25]. As a result, different autophagy features are expected between HCN and FN based on their differing tumor biology. Autophagy in HCN and FN has not been evaluated to date. In the present study, we evaluated the expression of autophagy-related proteins (beclin-1, LC3A, LC3B, p62, and BNIP3) in HCN and FN and assessed the clinical implications.

## 2. Materials and Methods

### 2.1. Patient Selection

Patients who underwent surgical resection and were diagnosed with FN and HCN at Severance Hospital between January 2000 and December 2013 were included. This study was approved by the Institutional Review Board of Yonsei University Severance Hospital. Patients who received preoperative chemotherapy were excluded. The histology of all resected specimens was retrospectively reviewed by a thyroid pathologist (JS Koo) with hematoxylin and eosin- (H&E-) stained slides. Clinicopathologic data were collected from the medical records and included age at diagnosis, recurrence, metastasis, death, follow-up period, tumor size, location (right or left lobe), tumor extent (intrathyroidal or extrathyroidal), and number of metastatic lymph nodes.

### 2.2. Tissue Microarray

Representative areas of hematoxylin-eosin-stained slides and corresponding spots were marked on the surface of the matching paraffin block. Three-millimeter cores were taken from selected areas of formalin-fixed, paraffin-embedded tissue (FFPE) and constructed into a 6 × 5 tissue microarray block. More than two tissue cores were extracted from each case, and each tissue core was assigned a unique tissue microarray location number that was linked to a database containing clinicopathologic data.

### 2.3. Immunohistochemistry

Antibodies used for immunohistochemistry are listed in Table 1. Sections from the TMA blocks were used for immunohistochemistry with an automatic immunohistochemistry staining device (Benchmark XT, Ventana Medical System, Tucson, AZ, USA). Briefly, 5 *μ*m thick FFPE sections were transferred onto adhesive slides and dried at 62°C for 30 minutes. Standard heat epitope retrieval was performed for 30 minutes in ethylene diamine tetra acetic acid, pH 8.0, in the autostainer. The samples were then incubated with primary antibodies, followed by biotinylated anti-mouse immunoglobulins, peroxidase-labeled streptavidin (LSAB kit, DakoCytomation), and 3,30-diaminobenzidine.

### 2.4. Interpretation of Immunohistochemical Staining

Immunohistochemical staining was evaluated by light microscopy. The stained slides were semiquantitatively evaluated according to a previously described method [26]. Tumor cell staining was assessed as 0, negative or weak immunostaining in <1% of the tumor/stroma; 1, focal expression in 1%–10% of the tumor; 2, positive in 11%–50% of the tumor; and 3, positive in 51%–100% of tumor. The whole tumor area was evaluated: a score 0-1 was considered negative and a score 2 or higher was considered positive. High autophagy status was defined as positive staining for three or more of the five autophagy-related proteins. Tumors were divided into high or low autophagy subtype, based on the autophagy status.

### 2.5. Protein Extraction from FFPE Tissues and Western Blot

Protein extractions from FFPE tissues were performed using a Qproteome FFPE tissue kit (Qiagen) according to the manufacturer's manual. Briefly, two or three sections from the same block were deparaffinized in xylene and rehydrated in a graded series of ethanol (100%, 96%, and 70%). The tissues were mixed with FFPE extraction buffer containing *β*-mercaptoethanol, incubated at 100°C for 20 min, at 80°C for 2 h with agitation at 750 rpm, and then centrifuged for 15 min at 14,000 ×g at 4°C. Extracted protein content in the supernatant was determined by Bradford assay (Bio-Rad). Equal amounts of protein from each sample extract were separated on SDS-PAGE gels and blotted onto nitrocellulose membranes (Bio-Rad). Western blotting was performed with primary antibodies against beclin-1. LC3A, LC3B, p62, BNIP3, and GAPDH (Abcam) and specific bands were detected using an ECL kit (GE Healthcare Life Sciences).

### 2.6. Statistical Analysis

Statistical analyses were performed using SPSS for Windows, Version 23.0 (SPSS Inc., Chicago, IL, USA). Student's *t*-test was used for continuous variables. Chi-square and Fisher's exact tests were used for categorical variables. Statistical significance was set at *P* < 0.05. Kaplan-Meier survival curves and log-rank statistics were employed to evaluate time to tumor recurrence and overall survival. Multivariate regression analysis was performed using the Cox proportional hazards model.

## 3. Results

### 3.1. Baseline Characteristics of Follicular Neoplasms and Hürthle Cell Neoplasms

There were 265 FNs comprised of 153 FAs and 112 FCs (99 minimally invasive types and 13 widely invasive types), and 108 HCNs comprised of 81 HCAs and 27 HCCs. Clinicopathologic features of FNs and HCNs are shown in Supplementary Tables 1 and 2 available online at https://doi.org/10.1155/2017/1372387, respectively.

### 3.2. Expression of Autophagy-Related Proteins in Follicular Neoplasms and Hürthle Cell Neoplasms

**Figure 1:**
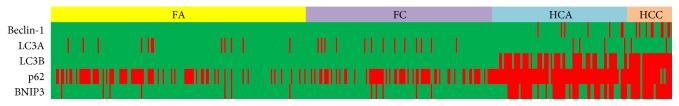
Heat map showing the expression of autophagy-related proteins in follicular neoplasms and Hürthle cell neoplasms. FA: follicular adenoma; FC: follicular carcinoma; HCA: Hürthle cell adenoma; HCC: Hürthle cell carcinoma; green: negative; red: positive.

**Figure 3:**
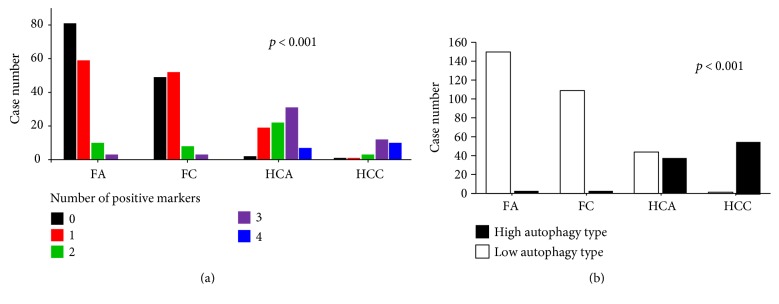
Number of positive autophagy-related proteins (a) and autophagy status (b) in follicular adenoma (FA), follicular carcinoma (FC), Hürthle cell adenoma (HCA), and Hürthle cell carcinoma (HCC). Positivity for various markers and high autophagy type are most frequently found in HCCs followed by HCAs, FCs, and FAs.

Expression of autophagy-related proteins in FNs and HNs is shown in Figures 1() and 2(). Expression of beclin-1, LC3B, p62, and BNIP3 was significantly higher in HCNs than in FNs (*P* < 0.001, Table 2). Expression of beclin-1, LC3B, and BNIP3 was highest in HCCs, followed by HCAs, FCs, and FAs (*P* < 0.001, Table 3). HCCs showed the highest positivity rate for autophagy-related proteins followed by HCAs, FCs, and FAs (*P* < 0.001). The frequency of high autophagy type (positive for three or more autophagy-related proteins) was highest in HCCs, followed by HCAs, FCs, and FAs (*P* < 0.001, Table 4, Figure 3()).

**Figure 4:**
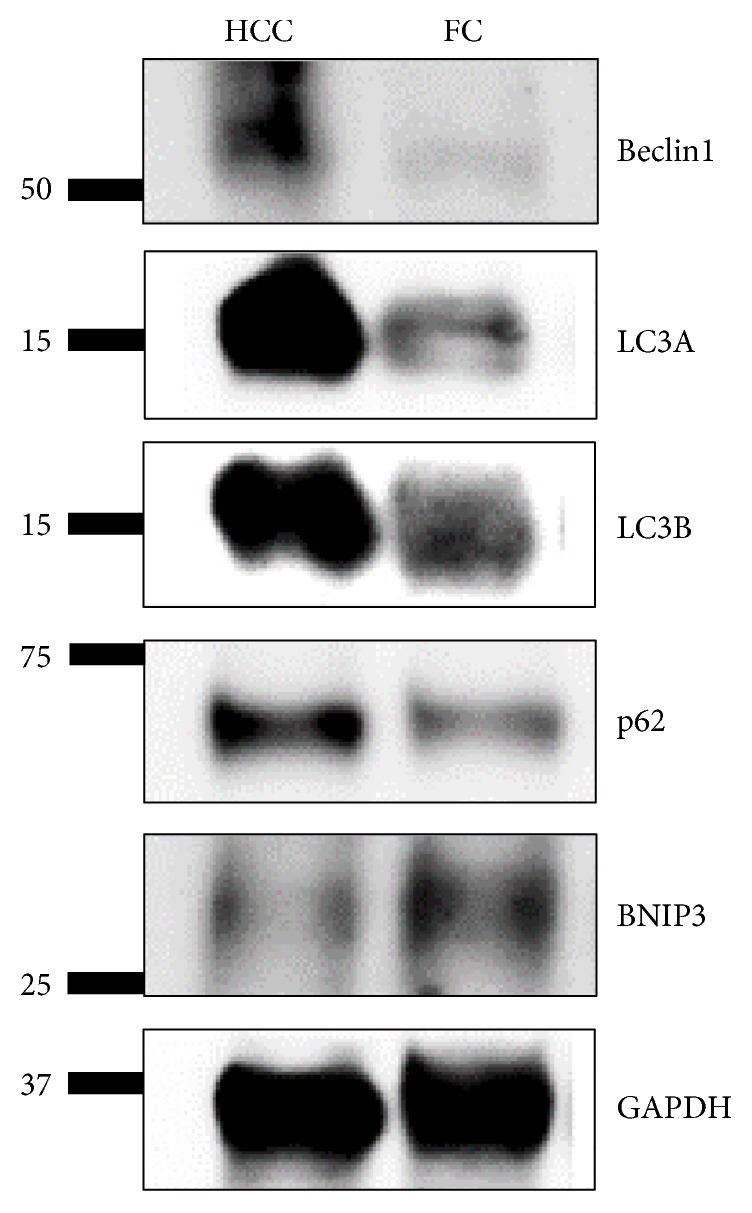
Western blot analysis for expression of autophagy-related proteins in follicular carcinoma (FC) and Hürthle cell carcinoma (HCC). Beclin-1, LC3A, LC3B, and p62 are more highly expressed in HCC than in FC.

Western blot analysis for autophagy-related proteins in HCC and FC revealed a higher expression of beclin-1, LC3A, LC3B, and p62 in HCC than in FC (Figure 4).

### 3.3. Correlation between the Expression of Autophagy-Related Proteins and Clinicopathologic Factors in Hürthle Cell Carcinomas and Follicular Carcinomas

**Figure 5:**
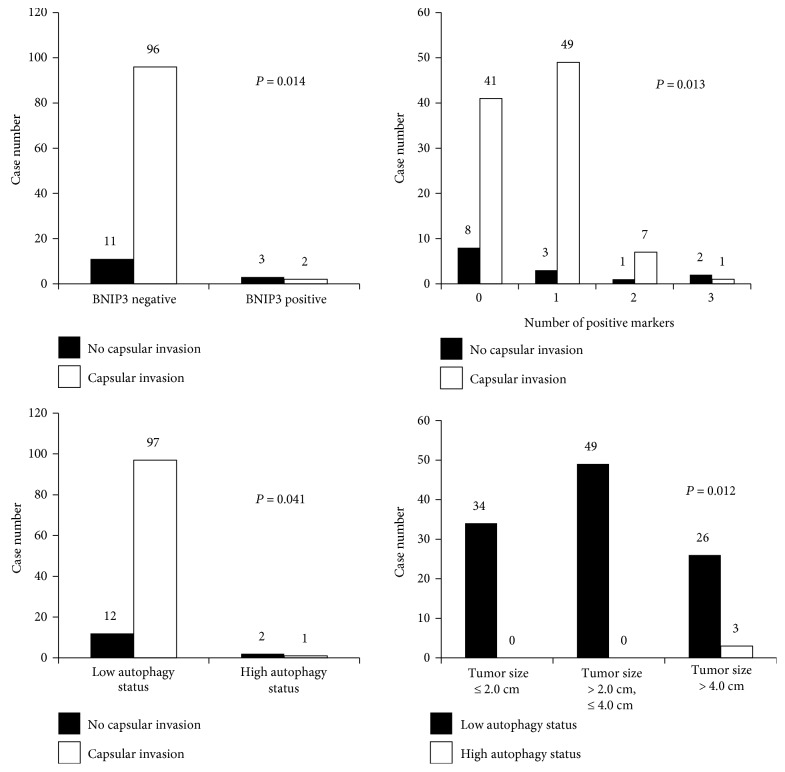
Correlation between the expression of autophagy-related proteins and clinicopathologic factors in follicular carcinoma.

There was no significant association between the expression of autophagy-related proteins and clinicopathologic factors in HCCs. In FCs, capsular invasion was associated with BNIP3 negativity (*P* = 0.014), low number of positive markers (*P* = 0.013), and low autophagy status (*P* = 0.041), and larger tumors (>4.0 cm) were associated with high autophagy status (*P* = 0.012 and Figure 5).

### 3.4. The Impact of Autophagy-Related Protein Expression on Prognosis

Expression of autophagy-related proteins had no significant effect on prognosis of FCs and HCCs (Table 5).

## 4. Discussion

We evaluated the expression of autophagy-related proteins in FNs and HCNs and observed that every autophagy-related protein was more highly expressed in HCNs than in FNs. Hürthle cells have an abnormally large number of mitochondria, which results in abundant acidophilic, granular cytoplasm. A previous study showed that XTC.UC1, a cell line derived from a Hürthle cell tumor, had increased autophagosome formation, which is consistent with the findings of the present study [27]. Biologically, Hürthle cells are less active than normal follicular epithelial cells [28], and increased autophagy activity appears to be linked to cellular functions of the Hürthle cell. Increased autophagy promotes degradation of ciliary proteins and reduces ciliogenesis [29]. These phenomena have been observed in Hürthle cells in lymphocytic thyroiditis, Hürthle cell carcinoma, and Hürthle cell variant papillary thyroid carcinoma [29]. Thus, regardless of the type of thyroid disease, Hürthle cells have increased autophagy activity, fewer ciliated cells, and shorter cilia. In this study, FCs and HCCs showed significantly higher expression of autophagy-related proteins compared to FAs and HCAs, which suggests higher autophagy activity in malignant tumors than in their benign counterparts. Expression of autophagy-related proteins is reportedly related to malignant progression in various tumors [30, 31], which is consistent with findings of the present study.

BNIP3, which is one of the mitophagy-related proteins, was more highly expressed in HCNs, as described in a previous study that showed activation of mitophagy in Hürthle cells. Mitophagy activation in Hürthle cells has been found to be ineffective [27], and abnormal mitochondria accumulate as a result of reduced turnover due to defective mitophagy caused by a *PARK2* gene mutation. We also found higher expression of BNIP3 in HCNs, but mitophagy itself may potentially be defective and further study is required.

One of the limitations of our study was that we used immunohistochemistry as an indicator of autophagy activity, which is a static method that may not be accurate since autophagy is more likely to be a multistep dynamic process. Since LC3A and LC3B are components of the autophagosome, expression of LC3A and LC3B could be interpreted as increased autophagy activation. However, an increased number of autophagosomes can be derived from delayed degradation, as well as under situations of increased autophagy activity. Therefore, monitoring of cellular autophagy flux at different time points is the most accurate for measuring autophagy activity [13]. However, we used paraffin blocks of tumor tissue and could not evaluate autophagy flux.

The other limitation of the present study is negative correlation between capsular invasion and expression of autophagy-related proteins in FC, which were partly incompatible with previous study and our conclusion that malignant tumor had higher autophagy activity. In the present study, HCC had overtly higher autophagy status than HCA and other FNs. Regarding FN, FC showed higher expression rate of autophagy-related proteins than FA, but most of FN encompassing FC and FA belonged to low autophagy status. Thus, it appears that autophagy-related proteins might not play an important role in tumor aggressiveness in FC compared to HCC. As seen in different distributions of recognized somatic mutations between FC and HCC, tumor aggressiveness like capsular invasion might be more dependent to factors other than autophagy-related proteins in FC [21].

Autophagy markers appear to be potential therapeutic targets in cancer therapy. Autophagy inhibition has been reported to suppress tumor growth in various tumors [32–35], and HCCs could be a target for autophagy inhibition considering its high expression of autophagy markers. In conclusion, expression of autophagy-related proteins (beclin-1, LC3B, p62, and BNIP3) was higher in HCNs and HCCs compared to FNs and HCAs, respectively, and this could have implications in cancer therapeutics.

## Supplementary Material

Supplementary Table 1. Basal characteristics of thyroid follicular carcinoma. Supplementary Table 2. Basal characteristics of Hürthle cell neoplasm.

## Figures and Tables

**Figure 2:**
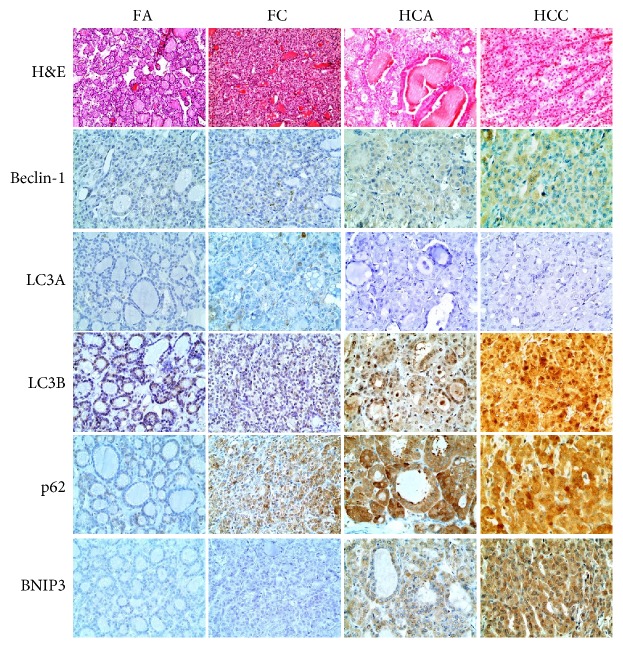
Expression of autophagy-related proteins in follicular adenoma, follicular carcinoma, Hürthle cell adenoma (HCA), and Hürthle cell carcinoma (HCC). Beclin-1, LC3B, p62, and BNIP3 are more highly expressed in Hürthle cell neoplasms than in follicular neoplasms and in HCCs than in HCAs.

**Table 1:** Source, clone, and dilution of antibodies.

Antibody	Clone	Dilution	Company
Beclin-1	Polyclonal	1 : 100	Abcam, Cambridge, UK
LC3A	EP1528Y	1 : 100	Abcam, Cambridge, UK
LC3B	Polyclonal	1 : 100	Abcam, Cambridge, UK
p62	SQSTM1	1 : 100	Abcam, Cambridge, UK
BNIP3	Ana40	1 : 100	Abcam, Cambridge, UK

**Table 2:** Expression of autophagy-related proteins in follicular neoplasms and Hürthle cell neoplasms.

Parameters	Total *N* = 373 (%)	Follicular neoplasm *N* = 265 (%)	Hürthle cell neoplasm *N* = 108 (%)	*P* value
Beclin-1				<0.001
Negative	357 (95.7)	265 (100.0)	92 (85.2)	
Positive	16 (4.3)	0 (0.0)	16 (14.8)	
LC3A				0.218
Negative	342 (91.7)	240 (90.6)	102 (94.4)	
Positive	31 (8.3)	25 (9.4)	6 (5.6)	
LC3B				<0.001
Negative	298 (79.9)	265 (100.0)	33 (30.6)	
Positive	75 (20.1)	0 (0.0)	75 (69.4)	
p62				<0.001
Negative	143 (38.3)	138 (52.1)	5 (4.6)	
Positive	230 (61.7)	127 (47.9)	103 (95.4)	
BNIP3				<0.001
Negative	294 (78.8)	253 (95.5)	41 (38.0)	
Positive	79 (21.2)	12 (4.5)	67 (62.0)	

**Table 3:** Expression of autophagy-related proteins in follicular adenomas, follicular carcinomas, Hürthle cell adenomas, and Hürthle cell carcinomas.

Parameters	Follicular neoplasm *N* = 265 (%)		Hürthle cell neoplasm *N* = 108 (%)		*P* value
	FA *N* = 153 (%)	FC *N* = 112 (%)	HCA *N* = 81 (%)	HCC *N* = 27 (%)	
Beclin-1					<0.001
Negative	153 (100.0)	112 (100.0)	74 (91.4)	18 (66.7)	
Positive	0 (0.0)	0 (0.0)	7 (8.6)	9 (33.3)	
LC3A					0.411
Negative	141 (92.2)	99 (88.4)	77 (95.1)	25 (92.6)	
Positive	12 (7.8)	13 (11.6)	4 (4.9)	2 (7.4)	
LC3B					<0.001
Negative	153 (100.0)	112 (100.0)	31 (38.1)	2 (7.4)	
Positive	0 (0.0)	0 (0.0)	50 (61.7)	25 (92.6)	
p62					<0.001
Negative	85 (55.6)	53 (47.3)	2 (2.5)	3 (11.1)	
Positive	68 (44.4)	59 (52.7)	79 (97.5)	24 (88.9)	
BNIP3					<0.001
Negative	146 (95.4)	107 (95.5)	37 (45.7)	4 (14.8)	
Positive	7 (4.6)	5 (4.5)	44 (54.3)	23 (85.2)	

FA: follicular adenoma; FC: follicular carcinoma; HCA: Hürthle cell adenoma; HCC: Hürthle cell carcinoma.

**Table 4:** Number of positive autophagy markers in follicular neoplasms and Hürthle cell neoplasms.

Number of positive markers for autophagy	Total *N* = 373 (%)	FA *N* = 153 (%)	FC *N* = 112 (%)	HCA *N* = 81 (%)	HCC *N* = 27 (%)	*P* value
0	133 (35.7)	81 (52.9)	49 (43.8)	2 (2.5)	1 (3.7)	<0.001
1	131 (35.1)	59 (38.6)	52 (46.4)	19 (23.5)	1 (3.7)	
2	43 (11.5)	10 (6.5)	8 (7.1)	22 (27.2)	3 (11.1)	
3	49 (13.1)	3 (2.0)	3 (2.7)	31 (38.3)	12 (44.4)	
4	17 (4.6)	0 (0.0)	0 (0.0)	7 (8.6)	10 (37.0)	

FA: follicular adenoma; FC: follicular carcinoma; HCA: Hürthle cell adenoma; HCC: Hürthle cell carcinoma.

**Table 5:** Univariate analysis of the influences of autophagy-related protein expression on disease-free and overall survival among patients with follicular carcinoma and Hürthle cell carcinoma (log-rank test).

Parameters	Number of patients/recurrence/death	Disease-free survival		Overall survival	
		Mean survival (95% CI) (months)	*P* value	Mean survival (95% CI) (months)	*P* value
Beclin-1			n/a		n/a
Negative	130/11/5	n/a		n/a	
Positive	9/0/0	n/a		n/a	
LC3A			0.942		0.359
Negative	124/10/4	115 (109–121)		120 (115–126)	
Positive	15/1/1	97 (81–112)		97 (81–112)	
LC3B			n/a		n/a
Negative	114/11/5	n/a		n/a	
Positive	25/0/0	n/a		n/a	
p62			0.104		0.406
Negative	56/7/3	88 (79–96)		95 (89–101)	
Positive	83/4/2	119 (113–125)		121 (113–128)	
BNIP3			n/a		n/a
Negative	111/11/5	n/a		n/a	
Positive	28/0/0	n/a		n/a	

CI: confidence interval; n/a: not applicable.
