# Supplementary material for: Expression of Autophagy-Related Proteins in Hürthle Cell Neoplasm Is Different from That in Follicular Neoplasm
Source: Dis Markers. 2017 Jul 27;2017:1372387. doi: 10.1155/2017/1372387 (PMC5551561; doi:10.1155/2017/1372387)
Supplement: Supplementary file 1 — Supplementary Table 1. Basal characteristics of thyroid follicular carcinoma. Supplementary Table 2. Basal characteristics of Hürthle cell neoplasm. [file 1372387.f1.docx]

| **Supplementary Table 1**. Basal characteristics of thyroid follicular carcinoma | | | | |
| --- | --- | --- | --- | --- |
| Parameters | Total  *N* = 112 (%) | FC, minimally invasive type  *N* = 99 (%) | FC, widely invasive type  *N* = 13 (%) | *P* - value |
| Age (years) |  |  |  | 0.255 |
| <45 | 51 (45.5) | 47 (47.5) | 4 (30.8) |  |
| ≥45 | 61 (54.5) | 52 (52.5) | 9 (69.2) |  |
| Sex |  |  |  | 0.233 |
| Male | 28 (25.0) | 23 (23.2) | 5 (38.5) |  |
| Female | 84 (75.0) | 76 (76.8) | 8 (61.5) |  |
| Tumor size (cm) |  |  |  | 0.040 |
| ≤2.0 | 34 (30.4) | 34 (34.3) | 0 (0.0) |  |
| >2.0, ≤4.0 | 49 (43.8) | 41 (41.4) | 8 (61.5) |  |
| >4.0 | 29 (25.9) | 24 (24.2) | 5 (38.5) |  |
| Capsular invasion |  |  |  | 0.147 |
| No | 14 (12.5) | 14 (14.1) | 0 (0.0) |  |
| Yes | 98 (87.5) | 85 (85.9) | 13 (100.0) |  |
| Vascular invasion |  |  |  | 0.028 |
| No | 66 (58.9) | 62 (62.6) | 4 (30.8) |  |
| Yes | 46 (41.1) | 37 (37.4) | 9 (69.2) |  |
| Tumor extension |  |  |  | <0.001 |
| Intrathyroidal | 95 (84.8) | 89 (89.9) | 6 (46.2) |  |
| Extrathyroidal | 17 (15.2) | 10 (10.1) | 7 (53.8) |  |
| LN metastasis |  |  |  | 0.220 |
| No | 110 (98.2) | 98 (99.0) | 12 (92.3) |  |
| Yes | 2 (1.8) | 1 (1.0) | 1 (7.7) |  |
| Distant metastasis |  |  |  | 0.003 |
| No | 101 (90.2) | 93 (93.9) | 8 (61.5) |  |
| Yes | 11 (9.8) | 6 (6.1) | 5 (38.5) |  |

FC, follicular carcinoma; LN, lymph node

**Supplementary Table 2**. Basal characteristics of Hürthle cell neoplasm

| Parameters | Total  *N* = 108 (%) | Hürthle cell adenoma  *N* = 81 (%) | Hürthle cell carcinoma  *N* = 27 (%) | *P* - value |
| --- | --- | --- | --- | --- |
| Age (years) |  |  |  | 0.310 |
| <45 | 45 (41.7) | 36 (44.4) | 9 (33.3) |  |
| ≥45 | 63 (58.3) | 45 (55.6) | 18 (66.7) |  |
| Sex |  |  |  | 0.136 |
| Male | 18 (16.7) | 16 (19.8) | 2 (7.4) |  |
| Female | 90 (83.3) | 65 (80.2) | 25 (92.6) |  |
| Tumor size (cm) |  |  |  | 0.005 |
| ≤2.0 | 76 (70.4) | 63 (77.8) | 13 (48.1) |  |
| >2.0, ≤4.0 | 20 (18.5) | 13 (16.0) | 7 (25.6) |  |
| >4.0 | 12 (11.1) | 5 (6.2) | 7 (25.9) |  |
| Capsular invasion |  |  |  | <0.001 |
| No | 84 (77.8) | 81 (100.0) | 3 (11.1) |  |
| Yes | 24 (22.2) | 0 (0.0) | 24 (88.9) |  |
| Vascular invasion |  |  |  | 0.002 |
| No | 105 (97.2) | 81 (100.0) | 24 (88.9) |  |
| Yes | 3 (2.8) | 0 (0.0) | 3 (11.1) |  |
| Tumor extension |  |  |  | <0.001 |
| Intrathyroidal | 102 (94.4) | 81 (100.0) | 21 (77.8) |  |
| Extrathyroidal | 6 (5.6) | 0 (0.0) | 6 (22.2) |  |
| LN metastasis |  |  |  | n/a |
| No | 108 (100.0) | 81 (100.0) | 27 (100.0) |  |
| Yes | 0 (0.0) | 0 (0.0) | 0 (0.0) |  |
| Distant metastasis |  |  |  | n/a |
| No | 108 (100.0) | 81 (100.0) | 27 (100.0) |  |
| Yes | 0 (0.0) | 0 (0.0) | 0 (0.0) |  |

LN, lymph node; n/a, not applicable
